# Low numeracy is associated with poor financial well-being around the world

**DOI:** 10.1371/journal.pone.0260378

**Published:** 2021-11-22

**Authors:** Wändi Bruine de Bruin, Paul Slovic

**Affiliations:** 1 Sol Price School of Public Policy and Dornsife Department of Psychology, University of Southern California, Los Angeles, California, United States of America; 2 Department of Psychology, Decision Research, University of Oregon, Eugene, Oregon, United States of America; University College London, UNITED KINGDOM

## Abstract

Numeracy refers to the ability to use numbers, including converting percentages (e.g., 10%) into absolute frequencies (e.g., 1 in 10). Studies have suggested that numeracy is correlated to financial outcomes, suggesting its relevance to financial decisions. However, almost all research on numeracy has been conducted in high-income countries in Europe and North America. Our analyses suggest that low numeracy is much more common in low-income countries, thus potentially threatening the financial well-being of the world’s poorest. We analyzed data from the Lloyd’s Register Foundation World Risk Poll, which assessed basic numeracy in 141 countries, including 21 low-income, 34 lower middle income, 43 upper middle income, and 43 high-income countries. Numeracy was associated with being among the poorest 20% of one’s country, and with difficulty living on one’s income, even after accounting for income, education, and demographics. These findings underscore the importance of worldwide numeracy education.

## Introduction

Numeracy refers to the ability to understand and use numbers [1]. Basic numeracy involves understanding how to convert simple percentages into frequencies, or recognizing that 10% is the same as 1 in 10 [2]. People are said to have “low numeracy” when they give an incorrect or no answer to such basic numerical problems. One study found that more than 30% of participants in the United States and Germany were unable to provide a correct answer to numeracy questions, though Germans did slightly better [3].

The Organisation for Economic Coordination and Development (OECD) [2] posits that numeracy is needed “to engage in and manage the mathematical demands of a range of situations in adult life.” Low numeracy can undermine people’s ability, confidence, and motivation to engage with numerical tasks [4–7]. Even after accounting for intelligence, low numeracy has been related to performing worse on decisions that involve numerical risks [8], even after accounting for intelligence [9]. Perhaps as a result, a Romanian study found that bankers with low numeracy have worse job performance [10].

There is ample evidence that low numeracy is associated with low income. Adults with low numeracy often have fewer employment opportunities and end up in lower paying jobs, according to national data from the United Kingdom [11]. A study in Australia suggested that lower education is associated with less numeracy and literacy, which in turn is associated with unemployment [12]. High-school graduates in Denmark who have not taken a math class report making less as compared to their peers who did take a math class [13]. In the United States, the Netherlands, and United Kingdom, people with low numeracy tend to report lower income and less wealth accumulation, even after accounting for education [6, 11, 14–16]. A Peruvian study also found that lower numeracy was associated with less wealth, assessed in terms of asset ownership (e.g., owning a fridge) and housing characteristics (e.g., toilet facilities.) [17].

Several studies from the United States also suggest that low numeracy may contribute to financial difficulties, independent of one’s income. Low-numerate adults are more likely to make actual financial mistakes, according to an analysis of consumer data that controlled for income [18]. Low numeracy has also been associated with taking on high-interest loans, failing to pay credit cards in full, and defaulting on mortgages, independent of income [19, 20]. Another study from the United States found that only 35% of participants had sufficient numeracy to correctly identify when a debt could never be paid off, after being told the amount owed on a credit card, the annual percentage rate, and monthly payments [21]. Additionally, community-dwelling older Americans with low numeracy may also be at greater risk of financial elder exploitation than their peers with high numeracy [22].

One main limitation of research on numeracy is that almost all studies have been conducted in countries in Europe and North America, which the World Bank has classified as high-income countries [23]. In high-income countries, lower educational attainment has been associated with low numeracy [2]. This raises a concern about the numeracy skills of people in low-income countries, who tend to go to school about half as many years as people in high-income countries [24]. Yet, numeracy may be more important to people in low-income countries, because they have to get by on a lot less money than in high-income countries. According to the World Bank’s classification, low-income countries are nations that have a per capita Gross National Income (GNI) of less than $1,026 [23]. By comparison, high-income countries have a GNI of more than $12,375 [23]. The middle income countries fall in between, with upper middle income countries having a GNI of $3,996-$12,375 and lower middle income countries having a GNI of $1,026-$3,995 [23].

The few country-comparisons that have been conducted have also been limited to high-income countries. The largest international comparison of numeracy ability was conducted through the OECD’s Survey of Adult Skills [2], but it was still limited to 39 countries that were mostly in the World Bank’s [23] high-income category. The study found that low numeracy skills affected 20% of participants aged 16–65 across the 39 participating countries [2]. It was as high as 50% in the few participating countries that were in the World Bank’s [23] upper middle income category, including Ecuador, Mexico and Peru. It may be even higher in countries from the World Bank’s [23] low-income and lower middle income category, which were missing from the OECD’s international comparison [2].

### The current paper

The current paper presents secondary analyses of the 2019 Lloyd’s Register Foundation World Risk Poll, which assessed the basic numeracy skills of more than 150,000 participants in 141 countries around the world, including 21 low-income countries, 34 lower-middle income countries, 43 upper-middle income countries, and 43 high-income countries [25].

Participants were asked a basic numeracy question: “Do you think that 10% is bigger than 1 out of 10, smaller than 1 out of 10, or the same as 1 out of 10?” The data were analyzed to assess (1) whether the percent of people with low numeracy differs between low-income, lower middle income, higher middle income and high-income countries; (2) whether low numeracy is associated with being among the poorest 20% of people in a country; (3) whether low numeracy is associated with difficulty making ends meet, even when accounting for income.

## Materials and methods

### Sample

The 2019 Lloyd’s Register Foundation World Risk Poll was conducted by Gallup and its local vendors [25]. The ethics committee at Gallup approved the survey, which complied with all ethical regulations. Numeracy was assessed among 153,165 participants in 141 countries and territories. According to the World Bank’s country classification [23], this sample included 21 low-income countries with a per capita gross national income (GNI) of less than $1,026, 34 lower middle income with GNI of $1,026-$3,995, 43 upper middle income countries with GNI of $3,996-$12,375, and 43 high-income countries with GNI of more than $12,375. Numeracy assessments were missing for high-income country Kuwait.

A national probability-based sample of approximately 1,000 people aged 15 or older was interviewed in each country or territory. Sample sizes were larger in China (N = 3709), India (N = 3,377), and Russia (N = 2,168) due to their large populations, and smaller in Jamaica (N = 501), due to its smaller population size. Participant characteristics appear in S1 Table. The data are publicly available from the UK Data Service, including a detailed description of the methodology (http://doi.org/10.5255/UKDA-SN-8739-1). Initial results were published in a report by Lloyd’s Register Foundation and Gallup [25].

Gallup used face-to-face interviews in 108 of the 141 countries, including all of the low-income countries, all of the lower-middle income countries, 40 of the 43 upper-middle income countries, and 13 of the 43 high-income countries. In Central and Eastern Europe, much of Latin America, former Soviet states, nearly all of Asia and the Middle East and Africa, Gallup used an area frame design for face-to-face interviewing. The coverage area is the entire country, including rural areas, and the sampling frame represents the entire non-institutionalized civilian population aged 15 and older. Sampling was stratified by population size and/or geography. Exceptions were made for areas that posed threats to the safety of interviewing staff, or scarcely populated islands that could only be reached by foot, animal or small boat.

Gallup conducted telephone surveys in 33 of the 141 countries and territories, where telephone coverage represents at least 80% of the population or is the customary survey methodology. This included 3 upper-middle income countries and 30 high-income countries. In those countries, sampling was based on probabilities proportional to population size. If such population statistics were unavailable, random sampling was used.

At least 30% of completed face-to-face interviews were validated using accompanied interviews, in-person re-contacts or telephone re-contacts. At least 15% of completed telephone interviews were validated by either listening to interviews live or listening to recorded interviews.

### Questions

Survey questions were asked in participants’ local language. They were translated into the major conversational languages of each country or territory, either through two independent translations or translation-backtranslation.

#### Numeracy

Participants were asked a basic numeracy question, performance on which is correlated with widely used multi-item numeracy measures [9]: “Do you think that 10% is bigger than 1 out of 10, smaller than 1 out of 10, or the same as 1 out of 10?” They were explicitly told that they could choose to skip the question: “If you do not know, please say so.” Responding that 10% is the same as 1 out of 10 was the correct answer. Low numeracy was defined as failing to provide that correct answer, either by stating that 10% is bigger or smaller than 1 out of 10, or giving no answer. Indeed, skipping the question has previously been counted as an incorrect response [4, 6].

#### Financial well-being

Interviewers asked participants about their monthly income before taxes in the currency of participants’ country of residence. To make the income variable comparable between countries and useful for global analyses, Gallup computed five similarly sized income categories for each country. These so-called “income quintiles” reflected whether each participant was among the 20% poorest people in their country, the 20% richest people in their country, or three income categories in between. A detailed description of this computation is publicly available (http://doi.org/10.5255/UKDA-SN-8739-1). Interviewers also asked participants “Which one of these phrases comes closest to your own feelings about your household’s income these days?”. Responses included whether “living comfortably on present income,” “getting by on present income,” “finding it difficult on present income” and “finding it very difficult on present income.”

#### Demographic variables

Demographic and control variables included participants’ education, age, and gender, because low numeracy has previously been associated with lower levels of education, female gender, and older age [4, 26]. The question about educational attainment referred to educational categories in each country, which reflected equivalency to having completed college, high school, or up to elementary school.

### Analyses

Analyses were conducted in SPSS version 26. Each research question was answered by conducting two analyses on the World Risk Poll dataset. The first analysis treated numeracy as a binary variable, with all participants who failed to give a correct answer being treated as having low numeracy, independent of whether they gave either of the two incorrect answers or no answer at all. The second analysis separately considered whether the participants who failed to provide the correct answer instead gave one of the two incorrect answers, or no answer at all.

These analyses were multilevel models that treated participants as nested within countries, using random intercepts for each country. They controlled for education (with dummies for whether or not participants had completed up to elementary school and high school), gender, and age. Age was divided by 10, thus reflecting decades rather than years, so that the associated odds ratios would be large enough to interpret. Interview mode was not included as a control variable, because it only varied in upper-middle income countries and high-income countries, and was therefore confounded with country income category (S1 Table). We did include interview mode in separate analyses for upper-middle income countries and high-income countries, but doing so did not affect overall conclusions (S2–S5 Tables). *P*-values below 0.05 were treated as significant. Exact *p*-values were provided for all values greater than or equal to 0.01. *P*-values less than 0.01 were referred to as *p* < .01 or *p* < 0.001 depending on which applied.

Our first research question asked whether the percent of people with low numeracy differed by World Bank country income category. To answer this research question, we conducted a multilevel binary logistic regression (Table 1; Model 1A) predicting low numeracy or failing to provide a correct answer to the basic numeracy question (vs. not). We also conducted a multilevel multinomial regression (Table 1; Model 1B) which separately considered whether participants who failed to provide a correct answer gave either of the two incorrect answers or no answer at all (vs. not). Predictor variables were dummy variables that reflected whether or not participants were recruited from a low income country, lower middle income country, or upper middle income country (vs. not), thus using high-income country as the comparison category.

**Table 1:** Odds ratios (95% confidence interval) from multilevel models predicting low numeracy.

	Model 1A: Incorrect or no answer (vs. correct answer)^a^	Model 1B: 10% is bigger than 1 in 10 (vs. correct answer)	Model 1B: 10% is smaller than 1 in 10 (vs. correct answer)		Model 1B: No answer (vs. correct answer)
***World Bank country income categories***					
**Low-income (vs. high-income)**	5.47***	7.69***	3.22***		5.61***
	(3.98, 7.50)	(5.22, 11.32)	(2.36, 4.40)		(3.83, 8.21)
	*p*<0.001	*p*<0.001	*p*<0.001		*p*<0.001
**Lower middle income (vs. high-income)**	3.54***	3.98***	2.44***		3.78***
	(2.57, 4.88)	(2.89, 5.47)	(1.87, 3.17)		(2.54, 5.63)
	*p*<0.001	*p*<0.001	*p*<0.001		*p*<0.001
**Upper middle income (vs. high-income)**	2.34***	2.51***	1.77***		2.46***
	(1.70, 3.22)	(1.79, 3.53)	(1.39, 2.24)		(1.65, 3.67)
	*p*<0.001	*p*<0.001	*p*<0.001		*p*<0.001
***Demographic variables***					
**Up to elementary school (vs college)**	4.30***	2.27***	2.09***	6.47***	
	(3.58, 5.17)	(1.80, 2.85)	(1.54, 2.83)	(5.07, 8.26)	
	*p*<0.001	*p*<0.001	*p*<0.001	*p*<0.001	
**High school (vs. college)**	2.11***	1.61***	1.27*	2.78***	
	(1.73, 2.56)	(1.28, 2.03)	(1.01, 1.60)	(2.24, 3.44)	
	*p*<0.001	*p*<0.001	*p* = 0.04	*p*<0.001	
**Female (vs. male)**	1.31***	1.03	1.20	1.44***	
	(1.16, 1.48)	(.88, 1.19)	(.99, 1.47)	(1.27, 1.63)	
	*p*<0.001	*p* = 0.75	*p* = 0.07	*p*<0.001	
**Age (divided by 10)**	1.16***	1.10***	1.09***	1.19**	
	(1.07, 1.26)	(1.05, 1.16)	(1.05, 1.13)	(1.07. 1.32)	
	*p*<0.001	*p*<0.001	*p*<0.001	*p* = 0.002	
***N***	151,712			151,712	
**Fixed effects ANOVA**	*F*(7, 151704) = 122.47***			*F*(21, 151688) = 118.34***	
**AIC**	5,350,468,634		15,998,386,296		
**BIC**	5,350,468,644		15,998,386,326		

^a^Low numeracy was defined as failing to provide a correct answer to the basic numeracy question, and giving one of the incorrect answers or no answer instead. *P*-values significant at ***p<0.001, **p<0.001, and *p<0.05. Model 1A represents multilevel logistic regression, Model 1B represents multilevel multinomial regression. Model fit was better for Model 1A than for Model 1B, seen in lower values of AIC = Akaike Information Criterion, corrected and BIC = Bayesian Information Criterion. According to the World Bank’s classification, low-income countries have a per capita gross national income of less than $1,026, lower middle income countries of $1,026-$3,995, upper middle income countries of $3,996-$12,375, and high-income countries of more than $12,375 [23].

Our second research question asked whether participants with low numeracy were more likely to be among the poorest 20% of people in a country. To answer this research question, we conducted two multilevel logistic regressions predicting whether or not participants were among the poorest 20% in their country of residence. Model 2A (Table 2) predicted this dependent variable from low numeracy or failing to provide a correct answer to the basic numeracy question (vs. not), and Model 2B (Table 2) predicted this dependent variable from whether or not participants gave either of the two incorrect answers or no answer to the basic numeracy question (vs. not).

**Table 2:** Odds ratios (95% confidence interval) from multilevel models predicting being among poorest 20% in country and reporting difficulty living on income.

	Being among poorest 20%		Difficulty living on income	
	Model 2A	Model 2B	Model 3A	Model 3B
***Low numeracy***				
**Incorrect or no answer (vs correct answer)**	1.70***	-	1.19***	-
	(1.33, 2.16)		(1.07, 1.32)	
	*p*<0.001		*p*<0.001	
**10% is bigger than 1 in 10 (vs. correct answer)**	-	1.45***	-	1.03
		(1.20, 1.76)		(0.91, 1.29)
		*p*<0.001		*p =* 0.69
**10% is smaller than 1 in 10 (vs correct answer)**	-	1.43***	-	1.09
		(1.25, 1.63)		(0.91, 1.29)
		*p*<0.001		*p* = 0.36
**No answer (vs. correct answer)**	-	1.83***	-	1.26***
		(1.39, 2.41)		(1.11, 1.42)
		*p*<0.001		*p*<0.001
***Demographic and control variables***				
**Poorest 20% (vs. richest 20%)**	-	-	6.59***	6.53***
			(4.85, 8.95)	(4.79, 8.89)
			*p*<0.001	*p*<0.001
**Second income quintile (vs. richest 20%)**	-	-	4.37***	4.35***
			(3.72, 5.13)	(3.71, 5.09)
			*p*<0.001	*p*<0.001
**Third income quintile (vs. richest 20%)**	-	-	2.73***	2.72***
			(2.22, 3.37)	(2.22, 3.33)
			*p*<0.001	*p*<0.001
**Fourth income quintile (vs. richest 20%)**	-	-	1.85***	1.84***
			(1.60, 2.14)	(1.60, 2.11)
			*p*<0.001	*p*<0.001
**Up to elementary school (vs. college)**	5.54***	5.41***	2.32***	2.28***
	(4.35, 7.06)	(4.29, 6.83)	(1.98, 2.71)	(1.97, 2.64)
	*p*<0.001	*p*<0.001	*p*<0.001	*p*<0.001
**High school (vs. college)**	2.21***	2.19***	1.54***	1.53***
	(1.77, 2.76)	(1.76, 2.72)	(1.39, 1.71)	(1.39, 1.69)
	*p*<0.001	*p*<0.001	*p*<0.001	*p*<0.001
**Female (vs. male)**	1.17*	1.16	0.96	0.95
	(1.01, 1.36)	(.99, 1.35)	(0.90, 1.02)	(0.89, 1.01)
	*p =* 0.04	*p =* 0.06	*p =* 0.17	*p =* 0.13
**Age (divided by 10)**	0.96	0.96	1.04	1.04
	(0.87, 1.06)	(0.87, 1.05)	(0.99, 1.10)	(0.99, 1.10)
	*p =* 0.40	*p =* 0.35	*p =* 0.13	*p =* 0.16
***World Bank country income categories***				
**Low-income (vs. high-income)**	0.38***	0.38***	7.08***	7.11***
	(0.33, 0.45)	(0.33, 0.45)	(4.83, 10.38)	(4.85, 10.42)
	*p*<0.001	*p*<0.001	*p*<0.001	*p*<0.001
**Lower middle income (vs. high-income)**	0.52***	0.52***	3.97***	3.96***
	(0.45, 0.60)	(0.44, 0.60)	(2.78, 5.68)	(2.77, 5.68)
	*p*<0.001	*p*<0.001	*p*<0.001	*p*<0.001
**Upper middle income (vs. high-income)**	0.64***	0.64***	2.66***	2.65***
	(0.57, 0.72)	(0.57, 0.72)	(1.96, 3.60)	(1.96, 3.60)
	*p*<0.001	*p*<0.001	*p*<0.001	*p*<0.001
***N***	150,634	150,634	147,544	147,544
**Fixed effects ANOVA**	*F*(8,	*F*(10,	*F*(12,	*F*(14,
	150625) = 63.05***	150623) = 65.28***	147531) = 162.38***	147529) = 167.43***
**AIC**	5,273,073,545	5,269,643,121	5,109,159,082	5,107,120,796
**BIC**	5,273,073,555	5,269,643,131	5,109,159,092	5,107,120,806

*P*-values significant at ***p<0.001, **p<0.001, and *p<0.05. Models represents multilevel logistic regression. Model fit was better for Model 2B than for Model 2A and for Model 3B than for Model 3A, seen in lower values of AIC = Akaike Information Criterion, corrected and BIC = Bayesian Information Criterion. According to the World Bank classification, low-income countries have a per capita gross national income of less than $1,026, lower middle income countries of $1,026-$3,995, upper middle income countries of $3,996-$12,375, and high-income countries of more than $12,375 [23]. Gallup computed income quintiles for each country, or five similarly sized income categories, include the 20% poorest people in their country, the 20% richest people in their country, and three income categories in between.

Our third research question asked whether low numeracy was associated with difficulty making ends meet, even when accounting for income. To answer this research question, we conducted two multi-level logistic regressions predicting whether or not participants had reported having difficulty living on their income. Model 3A (Table 2) predicted this dependent variable from low numeracy or failing to provide a correct answer to the basic numeracy question (vs. not), and Model 3B (Table 2) predicted this dependent variable from whether or not participants gave either of the two incorrect answers or no answer at all (vs. not). In addition to demographic variables, models 3A and 3B controlled for whether participants were among the poorest 20% (vs. richest 20%) in their country of residence, or whether they were in higher income quintiles (vs. richest 20%).

For all research questions, the Supplemental Materials show the results of the first model (Model 1A, 2A, and 3A), separately for each country income category (S2–S5 Tables). For the second and third research questions, the Supplemental Materials show analogous linear regression models that instead treated each dependent variable as a continuous variable, which did not affect the overall conclusions (Models 2C-D, Model 3C-D, S5 Table).

## Results

### Numeracy by country income category and demographic variables

**Fig 1:**
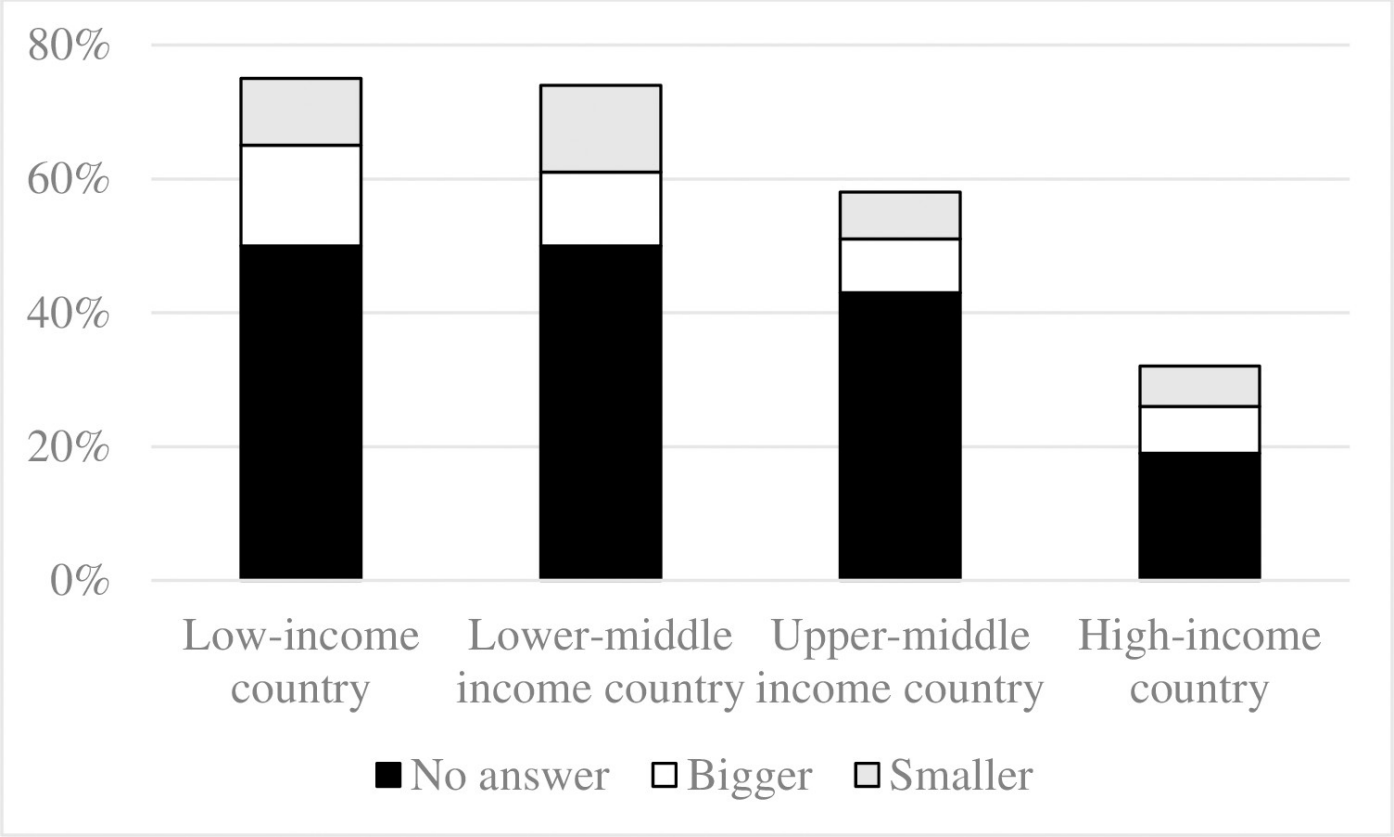
Percent of low-numerate participants by country income category. Note: According to the World Bank’s classification, low-income countries have a per capita gross national income of less than $1,026, lower middle income countries of $1,026-$3,995, upper middle income countries of $3,996-$12,375, and high-income countries of more than $12,375 [23].

Low numeracy, or failing to provide a correct answer to the basic numeracy question, varied from 76% in low-income countries and 73% in lower-middle income countries to 58% in upper-middle income countries and 32% in high-income countries (Fig 1). The percentage of low-numerate participants for each country is presented in S6 Table.

The percent of participants reporting a university degree varied by country income category, being 2% in low-income countries, 6% in lower middle income countries, 13% in upper middle income countries, and 28% in high-income countries (S1 Table). Yet, a multilevel logistic regression showed that differences in low numeracy by country income category held even after accounting for participants’ educational attainment and demographic characteristics (Table 1, Model 1A). Compared to participants from high-income countries, participants from low-income countries had about five times the odds of low numeracy, as seen in failing to provide the correct answer (vs. not) in response to the basic numeracy question (Table 1, Model 1).

**Fig 2:**
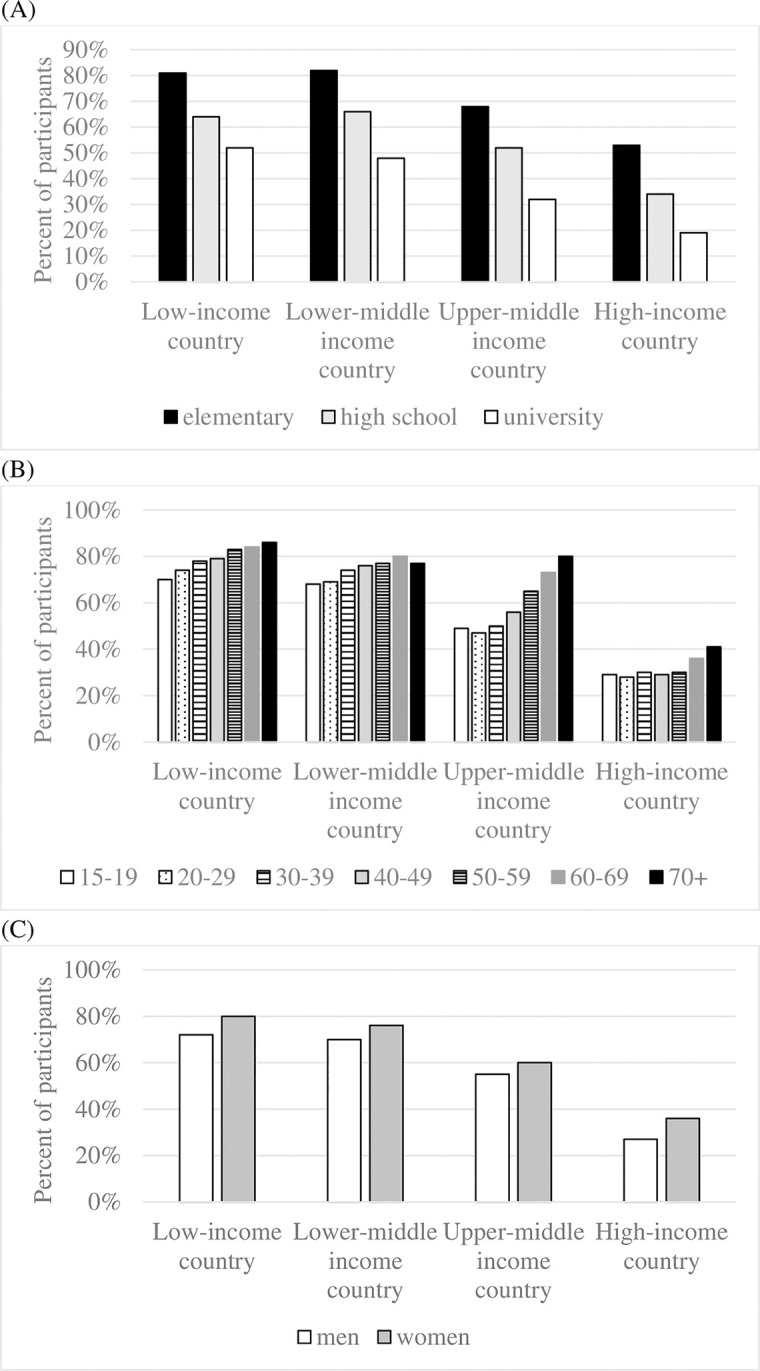
Percent of low-numerate participants by country income category and (A) educational attainment; (B) age and (C) gender. Note: According to the World Bank’s classification, low-income countries have a per capita gross national income of less than $1,026, lower middle income countries of $1,026-$3,995, upper middle income countries of $3,996-$12,375, and high-income countries of more than $12,375 [23].

Numeracy also tended to be lower among participants who reported lower levels of education, female gender, and older age, in models focused on 141 countries from across the world (Table 1) and on each country income category (S2 Table). Yet, living in a low-income country had the strongest association with low numeracy, as compared to lower education, older age, and female gender (Table 1, Model 1A). An examination of descriptive statistics further underscores the strong relationship between county income category and numeracy. For example, participants in high-income countries who had at most an elementary school education outperformed high-school graduates in countries in lower income categories, and performed on average about as well as university graduates in low-income countries (Fig 2A). As a second example, people aged 70+ from high-income countries on average outperformed participants of all ages from countries in lower income categories (Fig 2B). As a third example, women in high-income countries on average outperformed men and women in countries from lower income categories (Fig 2C).

Next, we further inspect the types of responses given by the 60% low-numerate participants who failed to accurately indicate that 10% was the same as 1 in 10. A majority declined to give an answer at all (42% of all participants), with the remainder stating that 10% was smaller than 1 in 10 (9% of all participants), or that 10% was bigger than 1 in 10 (9% of all participants). In each country income category, the latter two types of incorrect answers were similarly common (Fig 1). More importantly, in low-income countries, each type of incorrect answer was at least about twice as common as compared to high-income countries (Fig 1). For example, stating that 10% was bigger than 1 in 10 was observed among 15% of participants in low-income countries and 7% in high-income countries, stating that 10% was smaller than 1 in 10 was observed among 11% of participants in low-come countries and 6% of participants in high-income countries, and skipping the numeracy question was observed among 50% of participants in low-income countries and 19% of participants in high-income countries. A multinomial regression showed that these differences held even after accounting for participants’ demographic characteristics (Table 1, Model 1B).

### Relationship between low numeracy and low income

**Fig 3:**
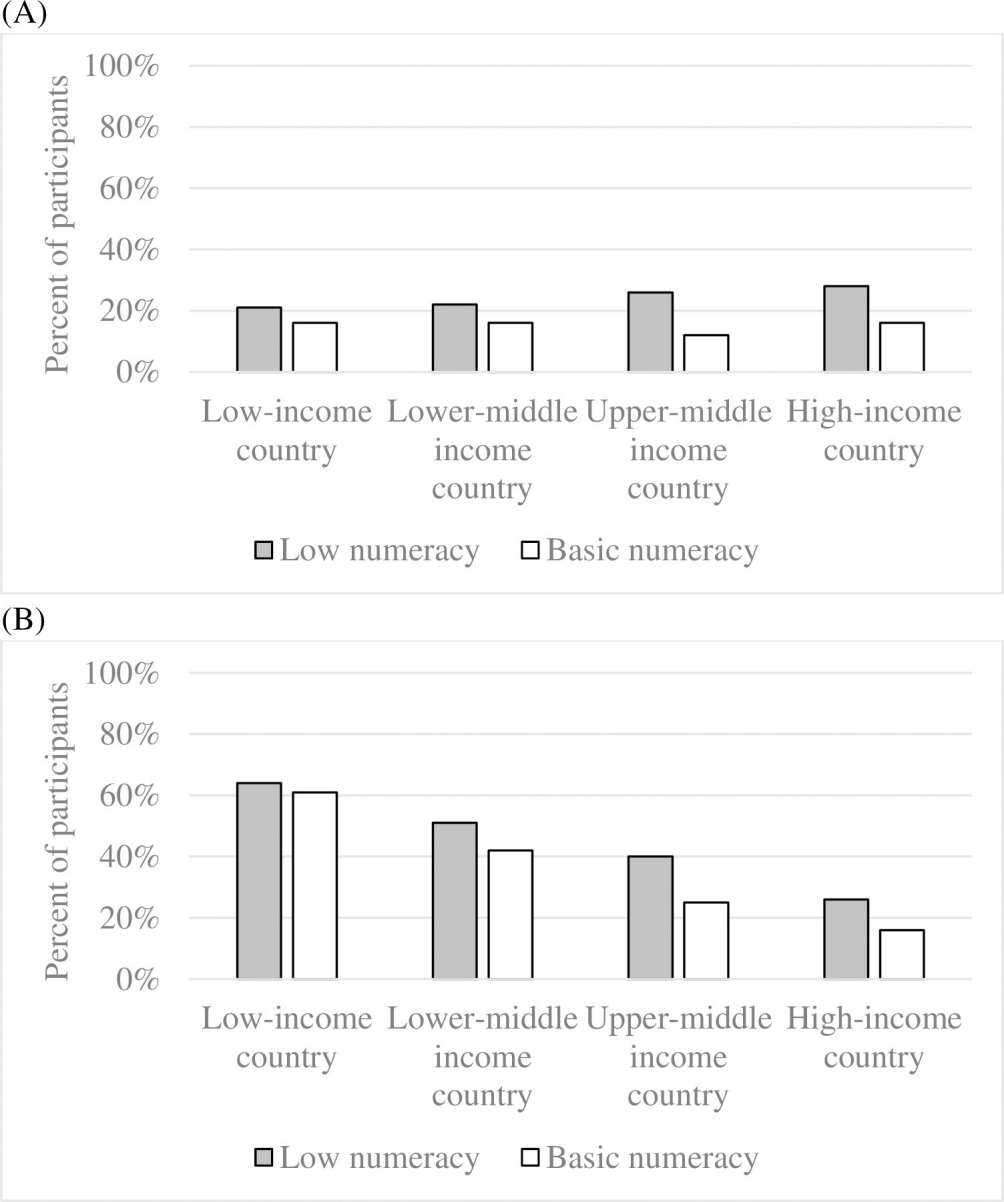
Percent of participants with low numeracy (vs. not) who reported (A) being among the poorest 20% in their country and (B) having difficulty living off their income. Note: According to the World Bank’s classification, low-income countries have a per capita gross national income of less than $1,026, lower middle income countries of $1,026-$3,995, upper middle income countries of $3,996-$12,375, and high-income countries of more than $12,375 [23].

The second set of analyses suggested that participants with low numeracy were more likely to be among the poorest 20% of people in their country of residence (Fig 3A). Around the world, 24% of participants with low numeracy reported income levels in the lowest quintile for their country of residence, while this was the case for 14% of participants who did answer the basic numeracy question correctly. Compared to individuals who answered the basic numeracy question correctly, those who did not give a correct answer had 70% greater odds of being among the poorest 20% of their country (Table 2, Model 2A). Additional multilevel multinomial regression analyses revealed that globally this finding was similarly driven by participants giving either of the two incorrect responses to the basic numeracy question and those who failed to answer the basic numeracy question at all (Table 2, Model 2B). The relationship between low numeracy and being among the poorest 20% in one’s country held in each World Bank country income category, and was the strongest in upper middle income countries (S3 Table).

The relationship between low numeracy and being among the poorest 20% in one’s country held despite controlling for educational attainment, which was a stronger correlate of being among the poorest 20% in one’s country (Table 2, Model 2A-B). Female gender and older age added little to nothing to models that predicted being among the poorest 20% in one’s country (Table 2, Model 2A-B).

### Relationship between low numeracy and difficulty making ends meet

The third set of analyses suggested that participants with low numeracy were more likely to indicate that they found it difficult to live on their income (Fig 3B). Around the world, 45% of participants with low numeracy reported finding it difficult to live on their income, as compared to 28% of participants with basic numeracy. The relationship between low numeracy and finding it difficult to live on one’s income held after accounting for whether or not participants’ income fell among the lowest 20% for their country, as well as education and demographic characteristics (Table 2, Model 3A). When considering whether low numerate participants provided an incorrect answer or no answer to the basic numeracy question, only the latter was significantly associated with difficulty living on one’s income (Table 2, Model 3B). Overall, low numeracy was associated with difficulty living on one’s income in all country income categories, except for low-income countries (S4 Table).

Closer inspection of control variables revealed that living in a low-income country and reporting low income were by far the strongest predictors of difficulty living on one’s income (Table 2, Model 3A-B). Participants who lived in low-income countries had about seven times the odds of reporting difficulty living on their income than those who lived in high-income countries, and participants who were among the poorest 20% in their country had about six times the odds of reporting difficulty living on their income than those who were among the richest 20% in their country (Table 2, Model 3A-B). Education also made a significant difference, such that participants with an elementary school education had twice the odds of reporting difficulty living on their income as compared to those who had a college education (Table 2, Model 3A-B). Female gender and did not additionally contribute to models predicting being among the poorest 20% in one’s country (Table 2, Model 3A-B).

## Discussion

Overall, the present findings suggest that low numeracy occurs around the world, but is much more common in low-income countries (76%) than in high-income countries (32%). Previous studies in high-income countries have suggested that low numeracy is most common among individuals who report lower levels of education, female gender, and older age [4, 26]. While our findings suggest these individual differences hold across the world, they also underscore that living in a low-income country is more strongly correlated with low numeracy.

People who were unable to make the simple numerical conversion between 10% and 1 in 10 tended to be among the poorest in their country, and had more difficulties living on their reported income. Thus, worldwide findings replicate conclusions that numeracy is associated with income and financial well-being, which were previously drawn on the basis of research in high-income countries [11–22]. All findings held in each World Bank country income category, with one exception. In low-income countries, reported difficulties living on one’s income were unrelated to low numeracy. Possibly, having basic numeracy skills may not help to overcome the greater financial struggles people face in low-income countries. Even people who are good with numbers will likely find it difficult to live comfortably on their income when there is simply not enough money. As noted, low-income countries have a per capita Gross National Income (GNI) of less than $1,026, while high-income countries have a GNI of more than $12,375 [23].

In addition to low numeracy, low levels of education were independently associated with participants’ reported income and difficulty living on one’s income. This is in line with research from Australia suggesting that education improves numeracy, literacy, and other skills that may contribute to employability [12]. However, it is important to note that low income was much more strongly associated with difficulty living on one’s income, as compared to numeracy and education. Specifically, participants in low-income countries were more likely to report difficulty living on their income than participants in high-income countries, and participants who were among the poorest 20% in their country were more likely to report difficulty living on their income than participants who were among the richest 20% in their country. These results suggest that policies that aim to promote financial well-being by improving numeracy and education will only go so far, and may need to be combined with economic support.

In this study, low numeracy referred to the inability to recognize that 10% is the same as 1 in 10. Low-numeracy was defined as answering that 10% is bigger than 1 in 10, 10% is smaller than 1 in 10, or declining to answer. A majority declined to give an answer at all (42% of all participants), with the remainder being equally likely to state that 10% was bigger or smaller than 1 in 10 (9% of all participants in each case). Previous studies in high-income countries have suggested that 1 in 10 might be viewed as larger than 10%, when receiving these numbers in vivid contexts [27–29]. For example, one study found that US participants felt that discharging a patient was perceived as more risky after reading that 1 in 10 (vs. 10%) of similar patients commit an act of violence to others during the first several months after discharge, likely due to the frequency format making it easier to imagine negative outcomes (e.g., ‘‘Some guy going crazy and killing someone” [30]. By comparison, World Risk Poll participants were asked about the similarity between 1 in 10 and 10% directly, without a specific context that could have swayed their imagination.

Like any study, ours has limitations. One limitation is that the World Risk Poll did not assess numeracy with the most commonly used multi-item numeracy measures [26, 31]. However, performance on the basic numeracy question in the World Risk Poll is known to be reliably correlated with traditional multi-item numeracy measures [9]. Indeed, single-item measures that correlate with multi-item measures can serve as valid predictors while reducing the cost and respondent burden of conducting a large global survey [32]. Another limitation is that no intelligence measures were included in the World Risk Poll. Due to the length of intelligence measures, doing so would likely would have been cost-prohibitive. Several previous studies have already demonstrated that intelligence and numeracy are correlated but separate constructs, that are both relevant for obtaining good financial outcomes [14, 16, 17, 19]. A third limitation is that the reported correlations do not necessarily imply causations. It is possible that people with higher incomes have jobs that help them hone their numerical skills, or that people who manage to live off their low income develop numeracy skills in the process of doing so.

To examine whether having basic numeracy skills causes better financial outcomes, studies are needed that randomly assign people to numeracy education and follow participants over time to examine the effect on their numeracy skills as well as their income and ability to live on it. One study in rural Ghana found that increasing access to education leads to better numerical skills, and, ultimately, better financial outcomes [33]. Another study in Colombia found that providing digital financial education in poor rural communities improved financial knowledge as well as financial well-being two years later, compared to controls [34]. Teaching numerical ability involves providing students with hands-on practical experience in a collaborative environment with regular assessments to monitor and repair understanding [35]. Numeracy education may need to improve both numerical ability and the confidence to apply that numerical ability [6]. Among American school children who had shown deficits and disinterest in mathematics, setting proximal achievable goals improved self-efficacy and numeracy performance [36]. Among undergraduate students enrolled in a psychology statistics course in the United States, numeracy could be improved through a values-affirmation intervention that may have prevented a decline in numerical confidence [37]. Such approaches show promise for tackling low numeracy across the world, which, when combined with much needed financial help, brings the promise of improving financial well-being.

## Supporting information

S1 Table Sample characteristics. (DOCX)

S2 Table Odds ratios (95% confidence interval) from multilevel models predicting low numeracy, for each World Bank country income category. (DOCX)

S3 Table Odds ratios (95% confidence interval) from multilevel models predicting being among the poorest 20% in one’s country of residence, for each World Bank country income category. (DOCX)

S4 Table Predictors of reporting difficulty living on income, by country income category. (DOCX)

S5 Table Standard estimates B (95% confidence interval) for multilevel linear regressions predicting income levels in quintiles for one’s country (1 = poorest; 5 = richest) and rated difficulty of living on one’s income (1 = living comfortably one present income; 4 = finding it very difficult on present income). (DOCX)

S6 Table Percent of participants with low numeracy in each country, by World Bank country income category. (DOCX)

## References

[1] Peters E. Innumeracy in the wild. Misunderstanding and misusing numbers. New York NY: New York University Press; 2020

[2] OECD, Skills Matter: Additional Results from the Survey of Adult Skills, OECD Skills Studies Publishing, Paris, 10.1787/1f029d8f-en; 2019

[3] Galesic M., Garcia-Retamero R. Statistical numeracy for health: A cross-cultural comparison with probabilistic national samples. Arch Int Med 2011; 170: 462–488 10.1001/archinternmed.2009.481 20212183

[4] Bruine de Bruin W, McNair SJ, Taylor AL, Summers B, Strough J. “Thinking about numbers is not my idea of fun”: Need for cognition mediates age differences in numeracy performance. Med Decis Making 2015; 35: 22–26 doi: 10.1177/0272989X14542485 25035261

[5] Fagerlin A, Zikmund-Fisher BJ, Ubel PA, Jankovic A, Derry HA, Smith DM. Measuring numeracy without a math test: Development of the subjective numeracy scale. Med Decis Making 2007; 27: 672–680 doi: 10.1177/0272989X07304449 17641137

[6] Peters E, Tompkins MK, Knoll MA, Ardoin SP, Shoots-Reinhard B, Meara AS. Despite high objective numeracy, lower numeric confidence relates to worse financial and medical outcomes. Proc Natl Ac Sci USA 2019; 116: 19386–19391 doi: 10.1073/pnas.1903126116 31501338 PMC6765274

[7] Skagerlund K, Lind T, Strömback C, Tinghög G, Västfjäll D. Financial literacy and the role of numeracy–How indivduals’ attitude and affinity with numbers influence financial literacy. J Behav Exp Econ 2018; 74: 18–25.

[8] Pachur T, Galesic M. Strategy selection in risky choice: The impact of numeracy, affect, and cross-cultural differences.” J Behav Decis Making 2013; 26: 260–71.

[9] Peters E, Västfjäll D, Slovic P, Mertz CK, Mazzocco K, Dickert. Numeracy and decision making. Psychol Sci 2006; 17: 407–413. doi: 10.1111/j.1467-9280.2006.01720.x 16683928

[10] Brown M, Kirschenmann K, Spycher T. Numeracy and on-the-job-performance: Evidence from loan officers. Econ Inquiry 2020; 58: 998–1022.

[11] Parsons S, Bynner J. Numeracy and employment. Educ + Train 1997; 39: 43–51.

[12] Chiswick BR, Lee YL, Miller PW. Schooling, literacy, numeracy, and labor market success. Econ Record 2003; 79: 165–181.

[13] Joensen JS, Nielsen HS. Is there a causal effect of high school math on labor market outcomes? J Human Resources 2009; 44: 171–198.

[14] Estrada-Meija C, de Vries M, Zeelenberg M. Numeracy and wealth. J Econ Psychol 2016; 54: 53–63

[15] Banks J, Oldfield Z. Understanding pensions: Cognitive function, numerical ability, and retirement saving. Fiscal Studies 2007; 28: 143–170

[16] Smith JP, McArdle J, Willis R. Financial decision making and cognition in a family context. Econ J. (Lond.) 2010; 120: F363–F380 10.1111/j.1468-0297.2010.02394.x PMC2992344 21116477

[17] Estrada-Meija C, Peters E., Dieckmann N.F., Zeelenberg M., de Vries, M., Baker M.P. Schooling, numeracy, and wealth accumulation: A study involving an argrarian population. J Cons Aff. 2020; 54:648–674.

[18] Agarwal S, Mazumder B. Cognitive Skills and Household Financial Decision Making. Am Econ J Appl Econ 2013; 5: 193–207.

[19] Sinayev A, Peters E. Cognitive reflection vs. calculation in decision making. Front. Psychol. 2015; 6: 532–547 doi: 10.3389/fpsyg.2015.00532 25999877 PMC4423343

[20] Gerardi K, Goette L, Meier S. Numerical ability predicts mortgage default. Proc Natl Ac Sci USA 2013; 110: 11267–11271. doi: 10.1073/pnas.1220568110 23798401 PMC3710828

[21] Lusardi A, Tufano P. Debt literacy, financial experiences, and overindebtedness. J Pension Econ Financ 2015; 14: 332–368

[22] Wood SA, Liu JP, Hanoch Y, Estevez-Cores S. Importance of numeracy as a risk factor for elder financial exploitation in a community sample. J Gerontol B Psychol Sci 2016; 71: 978–986. 10.1093/geronb/gbv041 26224756

[23] World Bank. New country classification by income level: 2019–2020; 2019. Available from: https://blogs.worldbank.org/opendata/new-country-classifications-income-level-2019-2020’

[24] Patrinos G, Psacharopoulos HA. Returns to investment in education: A decennial view of the global literature. Educ Econ 2018; 26: 445–458

[25] Lloyd’s Register Foundation, Gallup. The Lloyd’s Register Foundation World Risk Poll. Full report and analysis of the 2019 poll. London, UK: Lloyd’s Register Foundation, 2020. Available from: https://wrp.lrfoundation.org.uk/LRF_WorldRiskReport_Book.pdf

[26] Weller JA, Dieckmann NF, Tusler M, Mertz CK, Burns WJ, Peters E. Development and testing of an abbreviated numeracy scale: A Rasch analysis approach. J Behav Decis Making 2013; 26: 198–212 doi: 10.1002/bdm.1751 32313367 PMC7161838

[27] Monahan J, Heilbrun K, Silver E, Nabors E, Bone J, Slovic P. Communicating violence risk: Frequency formats, vivid outcomes, and forensic settings. International Journal of Forensic Mental Health 2012; 20: 121–126

[28] Sirota M, Juanchich M, Kostopoulou O, Hanak R. Decisive evidence on a smaller-than-you-think phenomenon: Revisiting the “1-in-X” effect on subjective medical probabilities. Med Decis Making 2014; 34: 419–429 doi: 10.1177/0272989X13514776 24310649

[29] Slovic P, Monahan J, MacGregor D.G. Violence risk assessment and risk communication: The effects of using actual cases, providing instruction, and employing probability versus frequency formats. Law and Human Behaviour 2002; 24: 271–296 10.1023/a:1005595519944 10846372

[30] Slovic P, Finucane M, Peters E, MacGregor DG. Risk as analysis and risk as feelings: Some thoughts about affect, reason, risk, and rationality. Risk Anal 2004; 24: 311–322 doi: 10.1111/j.0272-4332.2004.00433.x 15078302

[31] Cokely ET, Schulz E, Ghazal S, Garcia-Retamero R. Measuring risk literacy; The Berlin Numeracy Test. Judg Decis Making 2012; 7: 25–47

[32] Bergkvist L, Rossiter JR. The predictive validity of multiple-item versus single-item measures of the same constructs. J Market Research. 2007; 44: 175–184

[33] Peters E, Baker DP, Dieckmann NF, Leon J, Collins J. Explaining the effect of education on health: Psychol Sci 2010; 21: 1369–1376 doi: 10.1177/0956797610381506 20739672

[34] Attanasio O, Bird M, Cardona-Sosa L, Lavado P. Freeing financial education via tablets: Experimental evidence from Colombia. NBER working paper 25929. Available from: 10.3386/w25929

[35] Pearse M, Walton KM. Teaching numeracy: 9 critical habits to ignite mathematical thinking. Thousand Oaks CA: Corwin; 2011

[36] Bandura A, Schunk DH. Cultivating competence, self-efficacy, and intrinsic interest through proximal self-motivation. J Pers SocPsychol 1981; 41: 586–598

[37] Peters E, Shoots-Reinhard B, Tompkins MK, Schley MK, Meilleur L, Sinayev A, et al. Improving numeracy through values affirmation enhances decision and STEM outcomes. PLOS One 2017; 12: 1–19. doi: 10.1371/journal.pone.0180674 28704410 PMC5507517

