# Supplementary material for: Low numeracy is associated with poor financial well-being around the world
Source: PLoS One. 2021 Nov 22;16(11):e0260378. doi: 10.1371/journal.pone.0260378 (PMC8608299; doi:10.1371/journal.pone.0260378)
Supplement: S1 Table — (DOCX) [file pone.0260378.s001.docx]

S1 Table: Sample characteristics

|  | **World** | **Low-income countries** | **Lower middle income countries** | **Upper middle income countries** | **High-income countries** |
| --- | --- | --- | --- | --- | --- |
| ***Education*** | | | | | |
| **Up to elementary school (%)** | 44% | 72% | 49% | 52% | 10% |
| **High school (%)** | 43% | 25% | 45% | 34% | 61% |
| **University (%)** | 12% | 2% | 6% | 13% | 28% |
|  |  |  |  |  |  |
| ***Other demographic and control variables*** | | | | | |
| **Female (%)** | 51% | 52% | 49% | 51% | 51% |
| **Mean (SD) age** | 40.67 (17.49) | 33.82 (15.66) | 36.57 (15.97) | 42.27 (17.12) | 47.73 (18.77) |
| **Each income quintile** | 20% | 20% | 20% | 20% | 20% |
| **Face to face interview (vs. phone)** | 82% | 100% | 100% | 97% | 10% |

According to the World Bank’s classification, low-income countries have a per capita gross national income of less than $1,026, lower middle income countries of $1,026-$3,995, upper middle income countries of $3,996-$12,375, and high-income countries of more than $12,375 [24] Income quintiles reflect the poorest 20% in each country, the richest 20% in each country, and three equally sized income categories in between.
