# Supplementary material for: Low numeracy is associated with poor financial well-being around the world
Source: PLoS One. 2021 Nov 22;16(11):e0260378. doi: 10.1371/journal.pone.0260378 (PMC8608299; doi:10.1371/journal.pone.0260378)
Supplement: S2 Table — (DOCX) [file pone.0260378.s002.docx]

S2 Table: Odds ratios (95% confidence interval) from multilevel models predicting low numeracy, for each World Bank country income category.

|  | **Low-income country** | **Lower middle income country** | **Upper middle income country** | **High-income country** |
| --- | --- | --- | --- | --- |
| **Up to elementary school**  **(vs college)** | 3.15^***^  (1.74, 5.69)  *p*<0.001 | 3.76^***^  (2.74, 5.15)  *p*<0.001 | 4.21^***^  (3.25, 5.45)  *p*<0.001 | 4.67^***^  (3.28, 6.64)  *p*<0.001 |
| **High school**  **(vs. college)** | 1.51  (0.99, 2.31)  *p=*0.06 | 1.89^***^  (1.64, 2.19)  *p*<0.001 | 1.98^***^  (1.38, 2.82)  *p*<0.001 | 2.36^***^  (1.60, 3.47)  *p*<0.001 |
| **Female**  **(vs. male)** | 1.44^***^  (1.26, 1.64)  *p*<0.001 | 1.33^***^  (1.19, 1.49)  *p*<0.001 | 1.24^*^  (1.05, 1.46)  *p=*0.01 | 1.50^***^  (1.28, 1.74)  *p*<0.001 |
| **Age (divided by 10)** | 1.14^***^  (1.08, 1.20)  *p*<0.001 | 1.09^***^  (1.04, 1.13)  *p*<0.001 | 1.24^***^  (1.10, 1.39)  *p*<0.001 | 1.11^***^  (1.07. 1.15)  *p*<0.001 |
| **Face-to-face interview (vs. phone)** | - | - | 1.06  (0.47, 2.40)  *p*=0.88 | 1.06  (0.68, 1.66)  *p*=0.79 |
| ***N*** | 22,172 | 37,788 | 48,313 | 43,439 |
| **Fixed effects ANOVA** | *F*(4, 22167) = 29.71^***^ | *F*(4, 37783) = 45.32^***^ | *F*(5, 48307) = 417.84^***^ | *F*(5, 43433) = 66.88^***^ |
| **AIC** | 283,106,785 | 2,011,099,606 | 2,084,204,764 | 957,974,892 |
| **BIC** | 283,106,793 | 2,011,099,614 | 2,084,204,773 | 957,974,901 |

Low numeracy was defined as failing to provide a correct answer to the basic numeracy question, and giving one of the incorrect answers or no answer instead. *P*-values significant at ^***^*p*<0.001, ^**^*p*<0.001, and ^*^*p*<0.05. Models represent multilevel logistic regression. AIC=Akaike Information Criterion, corrected and BIC=Bayesian Information Criterion. According to the World Bank’s classification, low-income countries have a per capita gross national income of less than $1,026, lower middle income countries of $1,026-$3,995, upper middle income countries of $3,996-$12,375, and high-income countries of more than $12,375 [2]. Face-to-face interviews were conducted in all of the low-income countries, all of the lower-middle income countries, 40 of the 43 upper-middle income countries, and 13 of the 43 high-income countries.
