# Supplementary material for: Low numeracy is associated with poor financial well-being around the world
Source: PLoS One. 2021 Nov 22;16(11):e0260378. doi: 10.1371/journal.pone.0260378 (PMC8608299; doi:10.1371/journal.pone.0260378)
Supplement: S3 Table — (DOCX) [file pone.0260378.s003.docx]

S3 Table: Odds ratios (95% confidence interval) from multilevel models predicting being among the poorest 20% in one’s country of residence, for each World Bank country income category.

|  | **Low-income country** | **Lower middle income country** | **Upper middle income country** | **High-income country** |
| --- | --- | --- | --- | --- |
| **Low numeracy ^a^**  **(vs. not)** | 1.23^**^  (1.09, 1.40)  *p=*0.001 | 1.23^***^  (1.10, 1.38)  *p*<0.001 | 2.13^***^  (1.77, 2.56)  *p*<0.001 | 1.77^***^  (1.39, 2.26)  *p*<0.001 |
| **Up to elementary school**  **(vs. college)** | 5.78^***^  (2.79, 11.96)  *p*<0.001 | 3.99^***^  (2.97, 5.34)  *p*<0.001 | 5.78^***^  (4.03, 8.28)  *p*<0.001 | 5.72^***^  (3.33, 9.82)  *p*<0.001 |
| **High school**  **(vs. college)** | 3.51^***^  (1.67, 7.35)  *p<*0.001 | 1.45^*^  (1.02, 2.06)  *p*<0.001 | 2.46^***^  (1.84, 3.29)  *p*<0.001 | 2.28^***^  (1.79, 2.90)  *p*<0.001 |
| **Female**  **(vs. male)** | 1.23^**^  (1.08, 1.41)  *p=*0.001 | 1.26  (1.05, 1.52)  *p=*0.05 | 1.03  (0.92, 1.15)  *p=*0.58 | 1.36^***^  (1.13, 1.63)  *p<*0.001 |
| **Age (divided by 10)** | 1.00  (0.96, 1.03)  *p=*0.002 | 0.92^***^  (0.90, 0.95)  *p*<0.001 | 1.02  (0.88, 1.20)  *p=*0.78 | 0.89^*^  (0.80, 1.00)  *p=*0.04 |
| **Face-to-face interview (vs. phone)** | - | - | 0.87  (0.76, 1.00)  *p*=0.05 | 0.91  (0.69, 0.96)  *p*=0.02 |
| ***N*** | 22,172 | 37,788 | 47,235 | 43,439 |
| **Fixed effects ANOVA** | *F*(5, 22166) = 37.74^***^ | *F*(5, 37782) = 362.40^***^ | *F*(6, 47228) = 65.67^***^ | *F*(6, 43432) = 11.01^***^ |
| **AIC** | 282,068,544 | 1,997,506,620 | 2,005,889,326 | 962,913,854 |
| **BIC** | 282,068,552 | 1,997,506,629 | 2,005,889,335 | 962,913,862 |

Low numeracy was defined as failing to provide a correct answer to the basic numeracy question, and giving one of the incorrect answers or no answer instead. *P*-values significant at ^***^*p*<0.001, ^**^*p*<0.001, and ^*^*p*<0.05. Models represent multilevel logistic regression. AIC=Akaike Information Criterion, corrected and BIC=Bayesian Information Criterion. According to the World Bank classification, low-income countries have a per capita gross national income of less than $1,026, lower middle income countries of $1,026-$3,995, upper middle income countries of $3,996-$12,375, and high-income countries of more than $12,375 [24]. Face-to-face interviews were conducted in all of the low-income countries, all of the lower-middle income countries, 40 of the 43 upper-middle income countries, and 13 of the 43 high-income countries.
