# Supplementary material for: Low numeracy is associated with poor financial well-being around the world
Source: PLoS One. 2021 Nov 22;16(11):e0260378. doi: 10.1371/journal.pone.0260378 (PMC8608299; doi:10.1371/journal.pone.0260378)
Supplement: S4 Table — (DOCX) [file pone.0260378.s004.docx]

S4 Table: Predictors of reporting difficulty living on income, by country income category.

|  | **Low-income country** | **Lower middle income country** | **Upper middle income country** | **High-income country** |
| --- | --- | --- | --- | --- |
| **Low numeracy^a^**  **(vs. not)** | 1.00  (0.89, 1.13)  *p=*0.98 | 1.08  (0.98, 1.19)  *p=*0.11 | 1.24^*^  (1.04, 1.49)  *p=*0.02 | 1.27^***^  (1.14, 1.42)  *p<*0.001 |
| **Poorest 20%**  **(vs. richest 20%)** | 2.89^***^  (1.83, 4.56)  *p*<0.001 | 4.87^***^  (3.43, 6.92)  *p<*0.001 | 8.68^***^  (6.20, 12.14)  *p*<0.001 | 8.76^***^  (6.90, 11.12)  *p*<0.001 |
| **Second income quintile (vs. richest 20%)** | 2.73^***^  (1.98, 3.75)  *p*<0.001 | 4.38^***^  (3.45, 5.56)  *p<*0.001 | 5.07^***^  (4.18, 6.15)  *p*<0.001 | 4.04^***^  (3.22, 5.08)  *p*<0.001 |
| **Third income quintile (vs. richest 20%)** | 1.79^***^  (1.14, 2.82)  *p*<0.001 | 3.22^***^  (2.46, 4.22)  *p<*0.001 | 2.72^***^  (2.47, 2.99)  *p*<0.001 | 2.28^***^  (1.84, 2.82)  *p*<0.001 |
| **Fourth income quintile (vs. richest 20%)** | 1.57^***^  (1.27, 1.95)  *p*<0.001 | 2.03^***^  (1.71, 2.42)  *p<*0.001 | 1.83^***^  (1.64, 2.04)  *p*<0.001 | 1.60^***^  (1.38, 1.85)  *p*<0.001 |
| **Up to elementary school**  **(vs. college)** | 1.33  (0.76, 2.32)  *p=*0.32 | 2.88^***^  (2.53, 3.28)  *p*<0.001 | 1.89^***^  (1.46, 2.44)  *p*<0.001 | 2.47^***^  (1.51, 4.05)  *p*<0.001 |
| **High school**  **(vs. college)** | 0.99  (0.70, 1.41)  *p=*0.97 | 1.74^***^  (1.63, 1.87)  *p*<0.001 | 1.35^***^  (1.10, 1.64)  *p*=0.003 | 1.54^***^  (1.29, 1.85)  *p*<0.001 |
| **Female**  **(vs. male)** | 0.98  (0.86, 1.11)  *p=*0.70 | 0.98  (0.93, 1.03)  *p=*0.47 | 0.94  (0.83, 1.07)  *p=*0.37 | 0.91^*^  (0.82, 1.01)  *p=*0.06 |
| **Age (divided by 10)** | 1.06^*^  (1.00, 1.12)  *p=*0.04 | 1.01  (0.96, 1.06)  *p=*0.67 | 1.04  (0.91, 1.19)  *p=*0.54 | 1.10^***^  (1.07, 1.14)  *p*<0.001 |
| **Face-to-face interview (vs. phone)** | - | - | 0.72  (0.43, 1.22)  *p=*0.23 | 2.17  (1.32, 3.56)  *p<*0.01 |
| ***N*** | 21,608 | 36,887 | 46,208 | 42,841 |
| **Fixed effects ANOVA** | *F*(9, 21598) = 24.93^***^ | *F*(9, 36877)  = 289.80^***^ | *F*(10, 46197)  = 252.44^***^ | *F*(10, 42830)  = 189.63^***^ |
| **AIC** | 272,867,317 | 1,939,166,736 | 1,967,254,220 | 942,104,981 |
| **BIC** | 272,867,325 | 1,939,166,744 | 1,967,254,228 | 942,104,990 |

Low numeracy was defined as failing to provide a correct answer to the basic numeracy question, and giving one of the incorrect answers or no answer instead. *P*-values significant at ^***^*p*<0.001, ^**^*p*<0.001, and ^*^*p*<0.05. Models represents multilevel logistic regression. AIC=Akaike Information Criterion, corrected and BIC=Bayesian Information Criterion. According to the World Bank’s classification, low-income countries have a per capita gross national income of less than $1,026, lower middle income countries of $1,026-$3,995, upper middle income countries of $3,996-$12,375, and high-income countries of more than $12,375 [24]. Gallup computed income quintiles for each country, or five similarly sized income categories, including the 20% poorest people in their country, the 20% richest people in their country, and three income categories in between. Face-to-face interviews were conducted in all of the low-income countries, all of the lower-middle income countries, 40 of the 43 upper-middle income countries, and 13 of the 43 high-income countries.
