# Supplementary material for: Low numeracy is associated with poor financial well-being around the world
Source: PLoS One. 2021 Nov 22;16(11):e0260378. doi: 10.1371/journal.pone.0260378 (PMC8608299; doi:10.1371/journal.pone.0260378)
Supplement: S5 Table — (DOCX) [file pone.0260378.s005.docx]

S5 Table: Standard estimates B (95% confidence interval) for multilevel linear regressions predicting income levels in quintiles for one’s country (1=poorest; 5=richest) and rated difficulty of living on one’s income (1=living comfortably one present income; 4=finding it very difficult on present income)

|  | **Income quintile** | | **Rated difficulty living on income** | | |
| --- | --- | --- | --- | --- | --- |
|  | **Model 2C** | **Model 2D** | **Model 3C** | | **Model 3D** |
| ***Low numeracy*** |  |  |  | |  |
| **Incorrect or no answer (vs correct answer)** | -0.29^***^  (-0.39, -0.18)  *p*<0.001 | - | 0.04^***^  (1.07, 1.32)  *p*<0.001 | | - |
| **10% is bigger than 1 in 10**  **(vs. correct answer)** | - | -0.16^***^  (-0.26, -0.07)  *p*<0.001 | - | | -0.03  (-0.10, 0.05)  *p=*0.52 |
| **10% is smaller than 1 in 10**  **(vs correct answer)** | - | -0.19^***^  (-0.26, -0.13)  *p*<0.001 | - | | -0.03  (-0.14, 0.08)  *p*=0.59 |
| **No answer**  **(vs. correct answer)** | - | -0.34^***^  (-0.47, -0.22)  *p*<0.001 | - | | 0.07^***^  (0.03, 0.11)  *p*<0.001 |
| ***Demographic and control variables*** | | | | | |
| **Poorest 20%**  **(vs. richest 20%)** | - | - | 0.78^***^  (0.72, 0.83)  *p*<0.001 | | 0.77^***^  (0.71, 0.83)  *p*<0.001 |
| **Second income quintile (vs. richest 20%)** | - | - | 0.58^***^  (0.47, 0.70)  *p*<0.001 | | 0.58^***^  (0.47, 0.69)  *p*<0.001 |
| **Third income quintile (vs. richest 20%)** | - | - | 0.40^***^  (0.27, 0.53)  *p*<0.001 | | 0.40^***^  (0.27, 0.53)  *p*<0.001 |
| **Fourth income quintile (vs. richest 20%)** | - | - | 0.23^***^  (0.14, 0.31)  *p*<0.001 | | 0.23^***^  (0.14, 0.31)  *p*<0.001 |
| **Up to elementary school**  **(vs. college)** | -1.39^***^  (-1.51, -1.27)  *p*<0.001 | -1.37^***^  (-1.49, -1.26)  *p*<0.001 | 0.29^***^  (0.19, 0.39)  *p*<0.001 | | 0.28^***^  (0.19, 0.37)  *p*<0.001 |
| **High school**  **(vs. college)** | -0.68^***^  (-0.77, -0.59)  *p*<0.001 | -0.67^***^  (-0.76, -0.59)  *p*<0.001 | 0.13^***^  (0.09, 0.17)  *p*<0.001 | | 0.13^***^  (0.09, 0.16)  *p*<0.001 |
| **Female**  **(vs. male)** | -0.15^***^  (-0.24, -0.06)  *p<*0.001 | -0.14  (-0.24, -0.05)  *p<*0.01 | -0.01  (-0.02, 0.01)  *p=*0.60 | | -0.01  (-0.03, 0.01)  *p=*0.45 |
| **Age (divided by 10)** | 0.03  (-0.03, 0.09)  *p*=0.27 | 0.03  (-0.02, 0.09)  *p=*0.22 | 0.02  (0.00, 0.04)  *p=*0.03 | | 0.02  (0.00, 0.04)  *p=*0.05 |
| ***World Bank country income categories*** | | | | | |
| **Low-income**  **(vs. high-income)** | 0.73^***^  (0.63, 0.83)  *p*<0.001 | 0.73^***^  (0.63, 0.83)  *p*<0.001 | 0.80^***^  (0.66, 0.95)  *p*<0.001 | | 0.80^***^  (0.66, 0.95)  *p*<0.001 |
| **Lower middle income**  **(vs. high-income)** | 0.49^***^  (0.39, 0.59)  *p*<0.001 | 0.49^***^  (0.40, 0.59)  *p*<0.001 | 0.57^***^  (0.42, 0.71)  *p*<0.001 | | 0.57^***^  (0.42, 0.71)  *p*<0.001 |
| **Upper middle income**  **(vs. high-income)** | 0.33^***^  (0.25, 0.41)  *p*<0.001 | 0.33^***^  (0.25, 0.41)  *p*<0.001 | 0.39^***^  (0.27, 0.51)  *p*<0.001 | | 0.39^***^  (0.26, 0.51)  *p*<0.001 |
| ***N*** | 150,634 | 150,634 | 147,544 | 147,544 | |
| **Fixed effects ANOVA** | *F*(8,  150625) = 98.56^***^ | *F*(10,  150623) = 78.36^***^ | *F*(12,  147531) = 171.95^***^ | *F*(14,  147529) = 224.45^***^ | |
| **AIC** | 725,284 | 725,009 | 571,682 | 571,450 | |
| **BIC** | 725,304 | 725,029 | 571,702 | 571,470 | |

*P*-values significant at ^***^*p*<0.001, ^**^*p*<0.001, and ^*^*p*<0.05. Models represents multilevel logistic regression. Model fit was better for Model 2D than for Model 2C and for Model 3D than for Model 3C, seen in lower values of AIC=Akaike Information Criterion, corrected and BIC=Bayesian Information Criterion. According to the World Bank classification, low-income countries have a per capita gross national income of less than $1,026, lower middle income countries of $1,026-$3,995, upper middle income countries of $3,996-$12,375, and high-income countries of more than $12,375 [24]. Gallup computed income quintiles for each country, or five similarly sized income categories, include the 20% poorest people in their country, the 20% richest people in their country, and three income categories in between.
