# Supplementary material for: Low numeracy is associated with poor financial well-being around the world
Source: PLoS One. 2021 Nov 22;16(11):e0260378. doi: 10.1371/journal.pone.0260378 (PMC8608299; doi:10.1371/journal.pone.0260378)
Supplement: S6 Table — (DOCX) [file pone.0260378.s006.docx]

S6 Table: Percent of participants with low numeracy in each country, by World Bank country income category.

| **Low-income country** | | **Lower-middle income country** | | **Upper-middle income country** | | **High-income country** | |
| --- | --- | --- | --- | --- | --- | --- | --- |
| Afghanistan | 64% | Bangladesh | 74% | Albania | 46% | Australia | 22% |
| Benin | 75% | Bolivia | 75% | Algeria | 46% | Austria | 40% |
| Burkina Faso | 82% | Cambodia | 80% | Argentina | 58% | Bahrain | 51% |
| Chad | 86% | Cameroon | 64% | Armenia | 50% | Belgium | 11% |
| Ethiopia | 74% | Congo Brazzaville | 64% | Azerbaijan | 44% | Canada | 23% |
| Gambia | 87% | Egypt | 52% | Belarus | 30% | Chile | 59% |
| Guinea | 87% | El Salvador | 77% | Bosnia Herzegovina | 46% | Croatia | 27% |
| Liberia | 83% | Eswatini | 61% | Botswana | 80% | Cyprus | 38% |
| Madagascar | 63% | Ghana | 67% | Brazil | 70% | Denmark | 31% |
| Malawi | 88% | Honduras | 80% | Bulgaria | 44% | Estonia | 30% |
| Mali | 74% | India | 81% | China | 54% | Finland | 19% |
| Mozambique | 86% | Indonesia | 81% | Colombia | 82% | France | 35% |
| Nepal | 86% | Ivory Coast | 80% | Costa Rica | 74% | Germany | 42% |
| Niger | 84% | Kenya | 70% | Dominican Republic | 76% | Greece | 25% |
| Rwanda | 77% | Kyrgyzstan | 63% | Ecuador | 73% | Hong Kong | 33% |
| Sierra Leone | 89% | Laos | 93% | Gabon | 65% | Hungary | 36% |
| Tajikistan | 54% | Lesotho | 74% | Georgia | 56% | Ireland | 26% |
| Tanzania | 81% | Mauritania | 74% | Guatemala | 89% | Israel | 18% |
| Togo | 79% | Moldova | 54% | Iran | 41% | Italy | 36% |
| Uganda | 73% | Mongolia | 55% | Iraq | 52% | Japan | 33% |
| Yemen | 66% | Morocco | 68% | Jamaica | 63% | Latvia | 42% |
|  |  | Myanmar | 51% | Jordan | 37% | Lithuania | 43% |
|  |  | Nicaragua | 87% | Kazakhstan | 55% | Luxembourg | 42% |
|  |  | Nigeria | 70% | Kosovo | 40% | Malta | 47% |
|  |  | Pakistan | 73% | Lebanon | 18% | Netherlands | 20% |
|  |  | Palestine | 39% | Libya | 69% | New Zealand | 26% |
|  |  | Philippines | 47% | Malaysia | 64% | Norway | 34% |
|  |  | Senegal | 81% | Mauritius | 47% | Panama | 79% |
|  |  | Tunisia | 66% | Mexico | 76% | Poland | 36% |
|  |  | Ukraine | 38% | Montenegro | 38% | Portugal | 33% |
|  |  | Uzbekistan | 33% | Namibia | 65% | Saudi Arabia | 47% |
|  |  | Vietnam | 53% | North Macedonia | 50% | Singapore | 24% |
|  |  | Zambia | 84% | Paraguay | 71% | Slovakia | 28% |
|  |  | Zimbabwe | 48% | Peru | 80% | Slovenia | 42% |
|  |  |  |  | Romania | 47% | South Korea | 27% |
|  |  |  |  | Russia | 49% | Spain | 43% |
|  |  |  |  | Serbia | 30% | Sweden | 25% |
|  |  |  |  | South Africa | 74% | Switzerland | 46% |
|  |  |  |  | Sri Lanka | 56% | Taiwan | 33% |
|  |  |  |  | Thailand | 76% | U.A.E. | 59% |
|  |  |  |  | Turkey | 61% | U.K. | 32% |
|  |  |  |  | Turkmenistan | 14% | U.S. | 25% |
|  |  |  |  | Venezuela | 75% | Uruguay | 46% |
